# Adding capivasertib to fulvestrant in patients with hormone receptor-positive advanced breast cancer: a cost-effectiveness analysis

**DOI:** 10.3389/fphar.2024.1495082

**Published:** 2025-01-15

**Authors:** Yitian Lang, Qingqing Chai, Yan Lin, Bin Wu, Xiaoyan Liu

**Affiliations:** ^1^ Department of Pharmacy, Huangpu Branch, Shanghai Ninth People’s Hospital, Shanghai Jiao Tong University School of Medicine, Shanghai, China; ^2^ Department of Pharmacy, Shanghai Chest Hospital, Shanghai Jiao Tong University School of Medicine, Shanghai, China; ^3^ Department of Pharmacy, Huadong Hospital, Fudan University, Shanghai, China

**Keywords:** cost-effectiveness, HR-positive breast cancer, capivasertib plus fulvestrant, partitioned survival approach, economic evaluation

## Abstract

**Objective:**

Capivasertib, a novel pan-AKT inhibitor, shows significant antitumor activity against hormone receptor-positive advanced breast cancer. However, its cost-effectiveness of this treatment remains uncertain. This study aimed to evaluate the cost-effectiveness of capivasertib plus fulvestrant versus fulvestrant alone for advanced breast cancer treatment from the perspectives of healthcare payers in the United States. Meanwhile, a experimental analysis from the perspective of China, incorporating specific assumptions, was also conducted in this study.

**Methods:**

A partitioned survival model was constructed to project the progression of breast cancer. Overall survival (OS) and progression-free survival (PFS) data were obtained from the CAPItello-291 trial and extrapolated for long-term survival estimates. Direct medical costs and utility data were gathered. The primary outcome measure was incremental cost-utility ratio (ICUR) to evaluate the cost-effectiveness of treatment regimen. One-way sensitivity analyses (OWSA) and probabilistic sensitivity analyses (PSA) were conducted to assess the robustness of the results.

**Results:**

The base-case analysis estimated the ICUR for capivasertib plus fulvestrant versus fulvestrant alone to be $709,647 per quality-adjusted life-year (QALY) in the US. OWSA revealed that the results were sensitive to hazard ratio of OS and the cost of capivasertib. PSA demonstrated that capivasertib plus fulvestrant exhibited a 0% probability of cost-effectiveness in the US.

**Conclusion:**

Our finding suggests that, at its current price, capivasertib plus fulvestrant regimen is unlikely to be a cost-effective option compared to fulvestrant alone for HR-positive advanced breast cancer patients from the perspective of healthcare system in the US. For the experimental analysis based on specific assumptions from Chinese perspective, the therapy regimen was also found to lack cost-effectiveness.

## Introduction

Breast cancer poses a substantial disease burden and stands out as the principal contributor to cancer-associated mortality in the female population on a global scale. According to the latest GLOBOCAN 2022 estimates, approximately 2.3 million newly diagnosed cases and 0.66 million breast cancer-related deaths were recorded (Bray et al., 2024). Among these cases, the hormone receptor(HR) -positive and human epidermal growth factor receptor-2 negative (HER2-) breast cancer subtypes were the most prevalent, accounting for approximately 65%∼70% of all metastatic breast cancers (Giuliano et al., 2019; Babcock et al., 2020; Howlader et al., 2014). The growth of most HR-positive breast cancer cells is usually driven by ER (Lumachi et al., 2015). First-line treatment for advanced breast cancer of the estrogen receptor (ER)-driven subtype predominantly involves endocrine therapies, such as selective estrogen receptor modulator (SERM), selective estrogen receptor down-regulator (SERD) or aromatase inhibitors (AIs). These treatments are frequently complemented with cyclin-dependent kinase (CDK) 4/6 inhibitors. Despite their widespread use, a substantial number of patients develop resistance to CDK4/6 inhibitors and current endocrine therapies, leading to limited treatment alternatives (Lin et al., 2020). At present, breast cancer research is actively centered around optimizing endocrine therapy and devising strategies to overcome resistance in ER-driven breast cancer patients, addressing the entire spectrum of treatment stages. Several resistance mechanisms have been identified in the treatment of HR-positive and HER2-negative advanced breast cancers, among which is the overactivation of the phosphatidylinositol 3-kinase (PI3K)-AKT-PTEN pathway. This pathway is found to be altered in approximately 50% of these breast cancers (Pereira et al., 2016). Interestingly, even in patients with endocrine resistance, AKT signaling can be activated without the presence of genetic alterations in this pathway, indicating its significance in promoting cancer progression (Frogne et al., 2005). Therefore, understanding and targeting the PI3K-AKT-PTEN pathway have become crucial areas of research to improve therapeutic outcomes and overcome resistance in hormone receptor-positive breast cancer treatments (Miller et al., 2010; Hanker et al., 2020). Currently, PI3K and mTOR inhibitors are already approved in advanced breast cancer, while recently, AKT inhibitors have been recently developed as an innovative therapeutic approach (Andrikopoulou et al., 2022a). Capivasertib (AZD5363) is an orally bioavailable, small molecule inhibitor targeting all three AKT isoforms (AKT1, AKT2, and AKT3). Its potent and selective inhibitory activity on AKT results in the dephosphorylation of crucial downstream targets. Preclinical studies have shown significant antiproliferative effects of capivasertib in breast cancer cell lines (Davies et al., 2012; Ribas et al., 2015). Furthermore, its synergistic antitumor effects when combined with endocrine therapy underscore its potential as a promising treatment approach for hormone receptor-positive breast cancer. As an oral, ATP-competitive pan-AKT kinase inhibitor, capivasertib holds considerable promise for targeting the altered AKT signaling pathway, presenting new therapeutic opportunities for patients with advanced breast cancer who have progressed following prior aromatase inhibitor therapy, with or without a CDK4/6 inhibitor. This point has been substantiated by several clinical trials. The FAKTION study (NCT01992952), a phase 2 trial, revealed that adding capivasertib to fulvestrant compared with placebo plus fulvestrant extended the survival of participants with AI-resistant HR-positive, HER2-negative advanced breast cancer (Jones et al., 2020; Howell et al., 2022). The phase 3 CAPItello-291 study (NCT04305496) demonstrated that the combination of fulvestrant and capivasertib led to significantly prolonged progression-free survival (PFS) compared to treatment with fulvestrant alone (median PFS: 7.2 vs. 3.6, Hazard ratio: 0.6, 95% CI: 0.51–0.71, P < 0.001) in patients with HR-positive, HER2-negative advanced breast cancer who had experienced disease progression during or after previous aromatase inhibitor therapy with or without a CDK4/6 inhibitor (Turner et al., 2023). The positive outcome of these trials highlights the potential of combining fulvestrant and capivasertib as a promising treatment strategy for HR-positive breast cancer patients. Based on these satisfactory results, the FDA approved capivasertib in combination with fulvestrant for the treatment of HR-positive, HER2-negative locally advanced or metastatic breast cancer in November 2023 (Mullard, 2023; Drugs.com, 2023). Despite their efficacy, these novel treatment regimens are frequently accompanied by substantial price, imposing notable economic burden on patients and healthcare insurance systems. In this context, conducting cost-effectiveness analyses plays a pivotal role in assessing the clinical benefit of new interventions at a justifiable cost, thereby providing evidence and references for public health decision-making organization. Currently, the cost-effectiveness of capivasertib plus fulvestrant for patients with HR + advanced breast cancer is uncertain. Performing economic analyses to clarify the cost-effectiveness is meaningful and helpful for physicians, oncologists or healthcare decision-makers, particularly in situations where resources are limited.

Therefore, this study conducts a pharmacoeconomic evaluation of capivasertib in combination with fulvestrant therapy for HR + breast cancer from the perspective of the healthcare payers in the United States to provide essential economic evidence and references to facilitate informed decision-making. Meanwhile, an experimental analysis from the perspective of China, based on specific assumptions, was also performed to provide exploratory reference data for drug pricing upon its market entry.

## Material and methods

### Model structure

**FIGURE 1:**
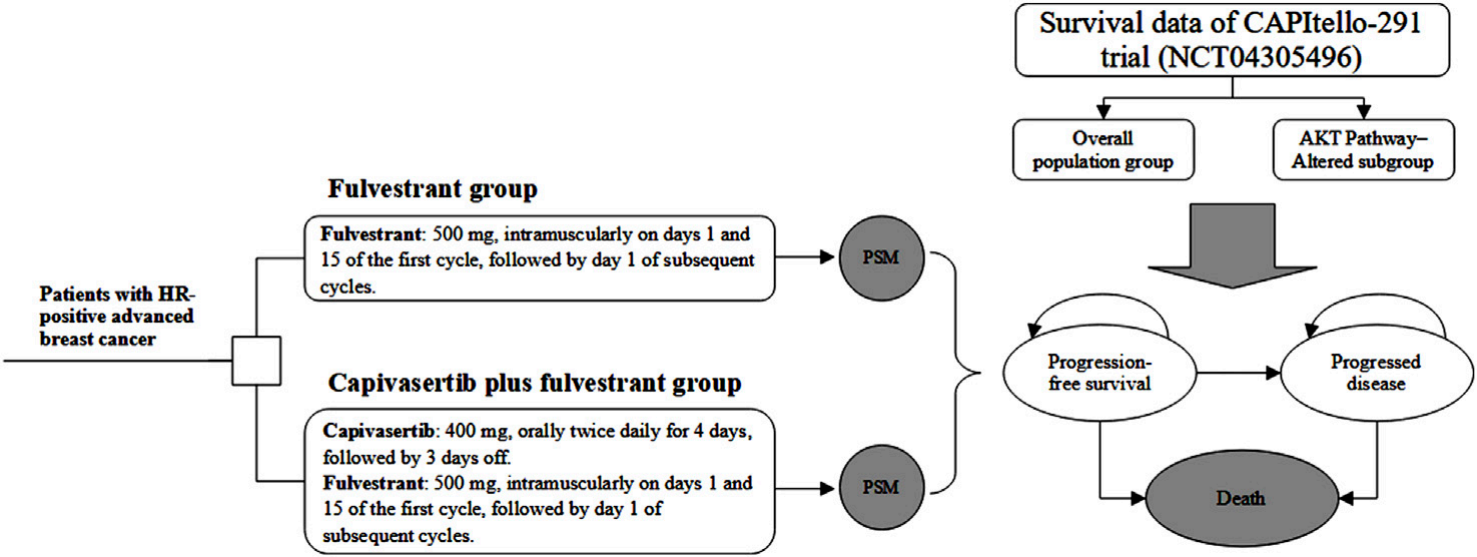
Study overview of economic evaluation of adding capivasertib to fulvestrant in hormone receptor-positive breast cancer. Notes: The model cycle is 4 weeks. Abbreviation: *PSM* partitioned survival model, *HR* hormone receptor.

We developed an economic evaluation model to compare the cost-effectiveness of capivasertib plus fulvestrant compared with placebo plus fulvestrant. A partitioned survival model (PSM) was constructed to simulate the progression of patients with HR-positive advanced breast cancer and incorporated three mutually exclusive health states, which includes progression-free (PF), progressed disease (PD) and death. The PF state was assumed as the default initial state, which could progress into either PD state or death state based on survival data. Through PSM, it is possible to directly determine the proportion of the cohort in each health state at any specific model time by utilizing the PFS and overall survival (OS) curves derived from clinical trials. A 4-week model cycle was used to facilitate cost estimates easier, which was in accordance with the administration cycle of capivasertib plus fulvestrant regimen. Regarding the time horizon for PSM, we are uncertain about the most suitable timeframe for analysis. Therefore, we have established three typical timeframes—five years, 10 years, and 15 years. For the base-case analysis, we chose the 10-year horizon. The decision tree and PSM structure are shown in Figure 1.

### Treatment regimens and resource use

This analysis incorporated treatment regimens based on the CAPItello-291 trial (Turner et al., 2023), including two interventions: 1) capivasertib in combination with fulvestrant as the treatment arm and 2) fulvestrant alone as the comparison arm. The dosing strategies for both groups were also in line with those applied in the CAPItello-291 trial. Therein, capivasertib was administrated orally at a dose of 400 mg twice daily for 4 days, followed by 3 days off. Fulvestrant was administrated intramuscularly at a dose of 500 mg on days 1 and 15 of the first cycle, followed by day 1 of subsequent cycles. All therapies continued until disease progression.

### Clinical data

In this analysis, some key clinical parameters including PFS and OS data and incidence of severe adverse events (AEs) were derived from the CAPItello-291 trial (Turner et al., 2023). The survival data consists of two cohorts, the (1) overall population group, and the (2) AKT pathway-altered subgroup, both of which will be included in the analysis of our study. Since the trial’s duration was insufficient for the current analysis of PSM timeframe, appropriate extrapolation beyond the trial’s follow-up period was required. Generally, prior to extrapolating curves, access to individual patient data (IPD) from clinical trials is required, followed by fitting it to the survival distribution. However, due to the limited public availability of IPD from clinical trials, we resort to obtaining pseudo IPD using a specific algorithm proposed by Guyot (Guyot et al., 2012). Currently, numerous survival analyses employ this approach to acquire IPD. We used the generated IPD of fulvestrant regimen to fit with several parametric distributions, including the Weibull, Log-logistic, Log-normal, exponential, Gompertz, and Gamma distributions (Jackson, 2016). The optimal survival distribution was chosen and validated through the Akaike Information Criterion (AIC) and visual inspection. If the distribution with the lowest AIC was selected but discrepancies were observed in the fitted curve compared to the original survival curve, particularly in the tail region indicating potential underestimation or overestimation of survival benefits, we employed a more flexible spline-based distribution, specifically the Royston/Parmar flexible parametric survival model, for that specific curve. This adjustment was implemented to ensure the utmost accuracy of values in the base-case analysis. The hazard ratio of capivasertib plus fulvestrant versus fulvestrant is used to generate the extrapolated curve of capivasertib plus fulvestrant regimen. Survival data used in this analysis were obtained from the survival data provided in the CAPItello-291 trial.

Treatment-related adverse events (AEs) were taken into account in the analysis. As grade 1 to 2 AEs are generally well-tolerated, our primary focus was on grade 3 and higher AEs. Table 1 contains detailed information on shape parameters of survival curve and the incidence of AEs.

**TABLE 1:** Key clinical data.

Groups	Parameters	Estimated values	Range	Distribution
Overall population group	PFS hazard ratio of capivasertib plus fulvestrant vs. placebo plus fulvestrant	0.60	0.51–0.71	Log-normal
	OS hazard ratio of capivasertib plus fulvestrant vs. placebo plus fulvestrant	0.74	0.56–0.98	Log-normal
	PFS: Placebo plus fulvestrant	gamma0 = −3.822	gamma0: −4.325 ∼ −3.319	Royston/Parmar spline model (2 knot)
		gamma1 = 7.080	gamma1: 5.683 ∼ 8.477	
		gamma2 = 1.427	gamma2: 1.046 ∼ 1.808	
		gamma3 = −0.979	gamma3: −1.284 ∼ −0.675	
	OS: Placebo plus fulvestrant	shape = 1.41050	Shape: 1.13292–1.75609 rate: 0.02516–0.05480	Gamma
		rate = 0.03713		
AKT pathway-altered subgroup	PFS hazard ratio of capivasertib plus fulvestrant vs. placebo plus fulvestrant	0.50	0.38–0.65	Log-normal
	OS hazard ratio of capivasertib plus fulvestrant vs. placebo plus fulvestrant	0.69	0.45–1.05	Log-normal
	PFS: Placebo plus fulvestrant	gamma0 = −4.124	gamma0: −5.017 ∼ −3.230	Royston/Parmar spline model (2 knot)
		gamma1 = 6.152	gamma1: 4.432–7.872	
		gamma2 = 2.578	gamma2: 1.651–3.506	
		gamma3 = −1.585	gamma3: −2.210 ∼ −0.960	
	OS: Placebo plus fulvestrant	meanlog = 3.379	meanlog: 3.038–3.720	Log-normal
		sdlog = 1.296	sdlog: 1.029–1.632	
Risk of main grade 3 and more AEs in the placebo plus fulvestrant group				
Diarrhea		0.3%	0.225%–0.375%	Beta
Rash		0.3%	0.225%–0.375%	Beta
Vomiting		0.6%	0.45%–0.75%	Beta
Anemia		1.1%	0.825%–1.375%	Beta
Risk of main grade 3 and more AEs in the capivasertib plus fulvestrant group				
Diarrhea		9.3%	6.975%–11.625%	Beta
Rash		12.1%	9.075%–15.125%	Beta
Vomiting		1.7%	1.275%–2.125%	Beta
Anemia		2.0%	1.5%–2.5%	Beta

*Abbreviations: AE* adverse event, *PFS* progression-free survival, *OS* overall survival.

### Costs and utilities

This analysis was carried out from the perspectives of the United States healthcare payers, with a specific focus on direct medical expenditures. These expenses encompassed various aspects, including therapy drugs, intramuscular injection administration, management of severe adverse events (AEs), follow-up care, subsequent treatment and end-of-life care. To obtain drug costs for the US perspective, data were collected from the Centers for Medicare and Medicaid Services (CMS), and the average sales price (ASP) reported by the manufacturers was adopted. Additionally, capivasertib price data were obtained from the Drugs.com website. To calculate the costs of intravenous injection administration, palliative care, follow-up visits, and subsequent treatment, we derived data from published studies or databases. Additionally, the expenses associated with managing severe AEs, specifically those graded as level 3 or higher, were gathered from relevant economic studies. To ensure comparability, all costs presented for years preceding 2023 were adjusted to 2023 using the Consumer Price Index (CPI).

In the context of cost-effectiveness analysis, health utility plays a vital role in computing cumulative quality-adjusted life-years (QALYs), which serve as a quantifiable measure of an individual’s health-related quality of life (HRQOL). Moreover, the quality of life is assumed to be associated with the progressive stages (PFS state or PD state) of patients with HR + advanced breast cancer, meaning that individuals receiving different treatment regimens at the same disease progression stage would have equivalent health utility values. Additionally, all costs and utilities were discounted, with an annual rate of 3% applied for the United States. Detailed input values are summarized in Table 2.

**TABLE 2:** Key model inputs Costs, Utility estimates and other parameters.

Parameter	Distribution	The US	
Treatment costs		Values (Range), USD	Reference
Capivasertib (per 200 mg)	Normal	$377.29 (282.97–471.61)	Truqap Prices (2024)
Fulvestrant (per 250 mg)	Normal	$87.26 (65.45–109.08)	Centers for Medicare & Medicaid Services (2024)
Administration (per cycle)	Normal	$702 (526.5–877.5)	Gauthier et al. (2018)
Follow-up	Normal	$2,959 (2,219.3–3,698.8)	Sorensen et al. (2012)
Subsequent treatment	Normal	$2,564 (1,923–3,205)	Rashid et al. (2016)
End-of-life care	Normal	$2,601 (1,950.8–3,251.3)	Sorensen et al. (2012)
AEs unit costs			
Diarrhea	Normal	$11,545 (8,658.8–14,431.3)	Elting and Shih (2004)
Rash	Normal	$6,577.7 (4,933.3–8,222.1)	Kuznik et al. (2022)
Vomiting	Normal	$3,905.7 (2,929.3–4,882.1)	Sorensen et al. (2012)
Anemia	Normal	$14,532 (10,899–18,165)	Elting and Shih (2004)

Utility estimates		Values (Range)	Reference
PFS state	Beta	0.837 (0.753–0.921)	Lloyd et al. (2006)
PD state	Beta	0.443 (0.399–0.487)	Lloyd et al. (2006)

Disutility estimates		Values (Range)	Reference
Diarrhea	Beta	−0.05 (−0.06 to −0.04)	Nafees et al. (2008)
Rash	Beta	−0.03 (−0.04 to −0.02)	Nafees et al. (2008)

In this table, the costs of AEs presented were paid on a per-event basis. All costs reported for years prior to 2023 are updated to 2023 using the CPI.

*Abbreviations: AEs* adverse events, *USD* US dollars ($), *CPI* Consumer Price Index.

### Analyses

In the base-case analysis, we conducted an assessment of the incremental cost-effectiveness ratio (ICER) to determine the additional cost per life-year (LY) gained between the two treatment regimens. Furthermore, the incremental cost-utility ratio (ICUR) was employed to evaluate the additional cost per quality-adjusted life-year (QALY). A regimen is considered “cost-effective” if the ICUR falls below the specified willingness-to-pay (WTP) threshold.

For the context of the United States, the health-benefit price benchmarks recommended by the Institute of Clinical and Economic Review typically range from $100,000 to $150,000 per QALY (Institute of Clinical and Economic Review, 2023). In this analysis, we adopted a threshold of USD 150,000 per QALY to assess the cost-effectiveness of the different treatment regimens within the United States.

To ensure the reliability of our findings, we conducted a series of uncertainty analyses, including one-way deterministic sensitivity analyses (DSA) and probabilistic sensitivity analyses (PSA). In the DSA, we examined the impact of individual input uncertainties on the incremental cost-utility ratio (ICUR). The annual discount rate ranged from 0% to 8%, while other model inputs were varied within the reported 95% confidence intervals (CI) or reasonable ranges (±25% of the base-case value). For the PSA, we employed Monte Carlo simulations with 1,000 iterations, simultaneously sampling key parameters based on pre-specified probability distributions. Costs were assigned normal distributions, and incidence rates of adverse events (AEs) and utilities were sampled with Beta distributions (Briggs et al., 2012). The detailed parameters of these probability distributions were summarized in Supplementary Table 1. The hazard ratio between the two therapy options was sampled using the log-normal distribution. To provide a comprehensive understanding of the treatment strategy’s cost-effectiveness at different thresholds, we generated cost-effectiveness acceptability curves (CEAC) and scatter plots. These visual representations allowed us to assess the likelihood of the treatment strategy being considered “cost-effective” across a range of thresholds. Additionally, scenario analyses were carried out. It was hypothesized that a Patient Assistance Program (PAP) policy was implemented or analyses were conducted with different timeframes to explore a potentially cost-effective price. All analyses, including PartSA and the cost-effectiveness model, were carefully implemented in R software.

Furthermore, our research also conducted an experimental analysis with specific assumptions in the context of China. The results and conclusions of this analysis only serve the preset conditions, and the relevant content can be found in Supplementary Content 1. It is hoped that it can provide certain economic data references for relevant researchers, pharmaceutical companies, and health decision-makers.

## Results

### Curve fitting

**FIGURE 2:**
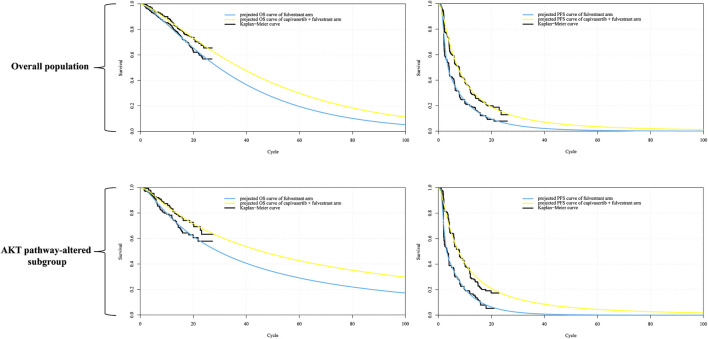
Reconstructed Kaplan-Meier survival curve and the projected OS and PFS curve. Notes: Each cycle of the x-axis is 4 weeks. Abbreviations: *KM* Kaplan-Meier; *PFS* progression-free survival, *OS* overall survival.

Using the Akaike Information Criterion (AIC), we initially screened for suitable survival distributions. In the overall population group, the PFS curve demonstrated the lowest AIC value when fitted with a lognormal distribution, while the OS curve exhibited the lowest AIC value when fitted with a gamma distribution. However, visual inspection revealed that although the lognormal distribution yielded the lowest AIC value for the PFS curve compared to other parameter distributions, it led to a notable underestimation of survival benefits in the curve’s tail. Recognizing that even the best-fitting distribution within the parameter model yielded suboptimal results, we employed the Royston/Parmar spline model (2 knots) for refitting, resulting in improved curve fitting. In the AKT pathway-altered subgroup, both the PFS and OS curves exhibited the lowest AIC values when fitted with the lognormal distribution. However, a similar situation of the PFS curve emerged through visual inspection, revealing suboptimal fit of the curves generated by the lognormal distribution. Consequently, we opted to utilize the Royston/Parmar spline model (2 knots) for refitting, resulting in improved curve fitting for PFS curves. As for the survival curve of the capivasertib plus fulvestrant regimen, it was generated through the principle of minimum AIC using the survival distribution combined with the Hazard Ratio. Upon visual inspection, it was found that the generated curve exhibited a good fit with the Kaplan-Meier curve, and no re-fitting adjustments were made. In other words, the PFS curve for capivasertib plus fulvestrant was derived by combining the lognormal distribution with the Hazard Ratio, rather than employing the Royston/Parmar spline model in conjunction with the Hazard Ratio. The replicated Kaplan-Meier survival curves and projected PFS and OS curves comparing the capivasertib plus fulvestrant to the placebo plus fulvestrant regimen were generated, as depicted in Figure 2. The curve parameters of the projected curve of the placebo plus fulvestrant arm can be found in Table 1.

### Base-case analysis

For the overall patient population, those receiving the fulvestrant regimen experienced a gain of 2.873 LYs, 1.415 QALYs, and incurred an expenditure of $183,767. In contrast, patients treated with the capivasertib plus fulvestrant regimen achieved 3.606 LYs, 1.845 QALYs, with a cost of $488,915. This indicates an incremental cost of $305,148 compared to the fulvestrant regimen. In terms of effectiveness, the capivasertib combination demonstrated an increase of 0.43 QALYs. The incremental cost-utility ratio (ICUR) of capivasertib plus fulvestrant versus fulvestrant was $709,647 per QALY.

Among patients with AKT pathway alterations, those on the fulvestrant regimen gained 3.551 LYs and 1.627 QALYs, incurring a cost of $224,779. Patients receiving the capivasertib plus fulvestrant regimen experienced gains of 4.699 LYs and 2.266 QALYs, with costs amounting to $563,274. This resulted in an incremental cost of $338,319 and an increase of 0.639 QALYs. The ICUR for the capivasertib plus fulvestrant regimen versus fulvestrant was $529,726 per QALY.

### One-way sensitivity analysis

**FIGURE 3:**
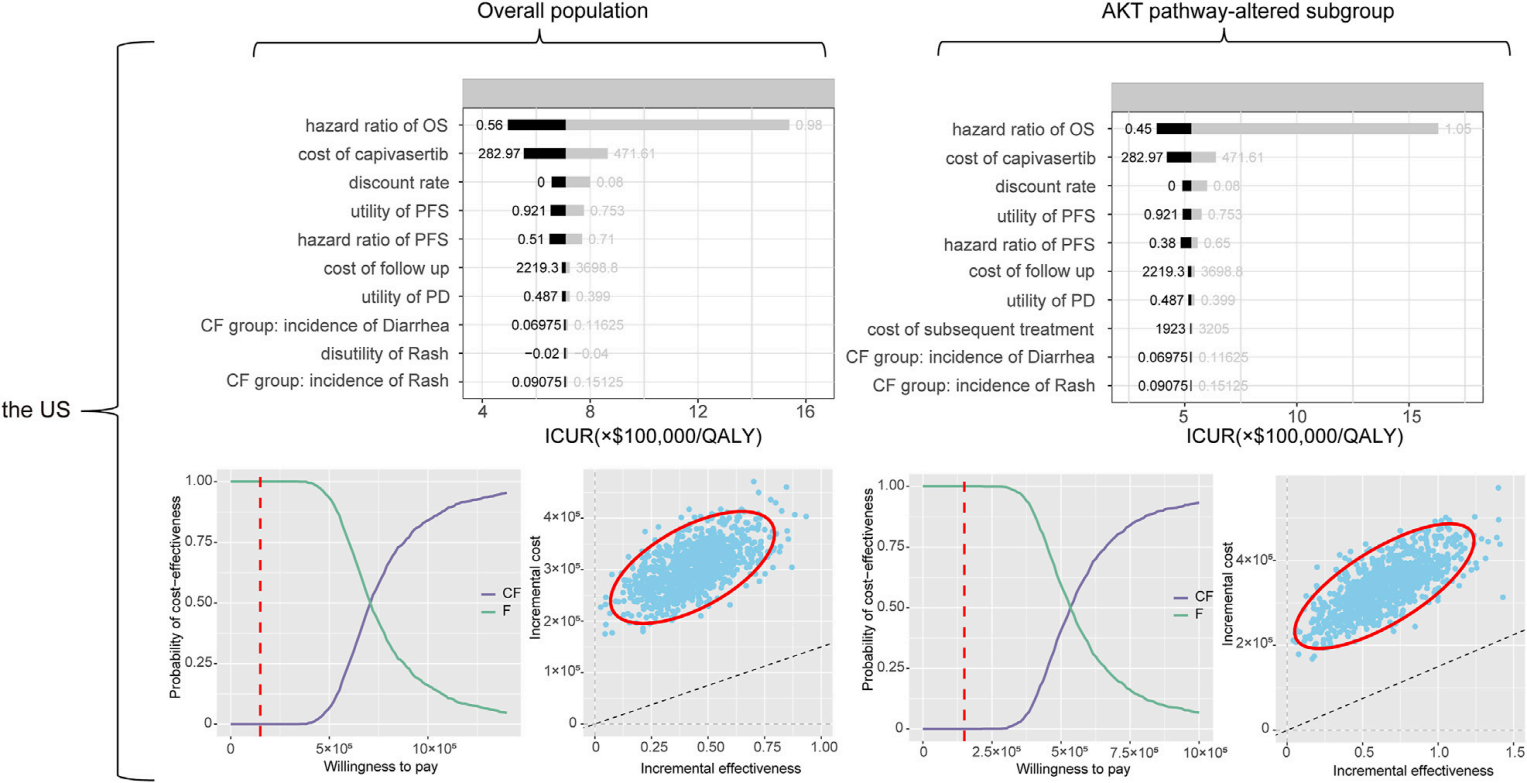
The output of the one-way sensitivity analysis and probabilistic sensitivity analysis. Notes: In the incremental cost-effectiveness scatter plot, each point represents one output. The red circle denotes the 95% confidence ellipse, while the black dashed line signifies the WTP threshold. In the cost-effectiveness acceptability curve, the y-axis shows the probability that a regimen is cost-effective at different willingness-to-pay thresholds (x-axis). The red dashed line represents the WTP threshold. The monetary unit of the WTP threshold is the United States dollar. Abbreviations: *CF* capivasertib plus fulvestrant regimen, *F* fulvestrant regimen alone, PFS progression-free survival, PD progressed disease, ICUR incremental cost-utility ratio, QALY quality-adjusted life-year.

The tornado diagrams, shown in Figure 3, were generated to facilitate the interpretation of key input variables impacting the analysis results.

In overall patient population, the diagram of tornado revealed that the hazard ratio of OS, cost of capivasertib, discount rate, utility of PFS, and hazard ratio of PFS were the key driving factors significantly impacting the ICUR between capivasertib plus fulvestrant and fulvestrant regimen alone. The range of the ICUR varied from $ 494,439/QALY to $ 1,538,648/QALY. In the AKT pathway-altered subgroup, the similar sensitivity factors as in the overall patient population were present. The hazard ratio of OS, cost of capivasertib, discount rate, utility of PFS, and hazard ratio of PFS were the key determinants significantly influencing the ICUR between the capivasertib plus fulvestrant regimen and the fulvestrant regimen alone. The ICUR ranged from $ 374,738 per QALY to $ 1,630,733 per QALY. In both the overall population group and the AKT pathway-altered subgroup, reducing the cost of capivasertib and the hazard ratio of capivasertib plus fulvestrant versus fulvestrant in terms of OS contributed to a decrease in the ICUR.

### Probabilistic sensitivity analysis

The outcomes, derived from the simultaneous extraction of all model parameters via probabilistic sampling, were graphically represented through CEAC and scatter diagrams, as illustrated in Figure 3.

The CEAC for the overall population indicated that the combination of capivasertib and fulvestrant regimen has an almost 0% probability of being cost-effective at a threshold of $150,000/QALY. In contrast, the fulvestrant regimen alone exhibited nearly 100% probability of cost-effectiveness at the same threshold. In the subgroup with AKT pathway alterations, the capivasertib plus fulvestrant regimen again demonstrated almost 0% probability of being cost-effective, while the fulvestrant regimen alone remained nearly 100% probability of cost-effectiveness.

### Scenario analysis

Capivasertib, a novel medication, has recently been approved for use in the US, but it comes with a high price tag. To alleviate the financial burden for eligible patients, the pharmaceutical company has launched a PAP. PAPs are initiatives that help uninsured and underinsured individuals access affordable medications. Typically administered by pharmaceutical companies, nonprofit organizations, or government bodies, these programs can either fully cover the cost of medications or provide them at a discounted rate. In the scenario analysis, we focus on the patient’s perspective and only the direct medical expenditures are considered. This drug is listed under Medicare Part D, allowing qualified patients to access the treatment at the lowest possible cost. Consequently, we conducted the scenario analysis on time horizon and the pricing of capivasertib to explore potential cost-effective pricing strategies. All results were summarized in the Table 3. Furthermore, based on the economic analysis model, we estimated the maximum price at which the drug can achieve cost-effectiveness thresholds ($150,000/QALY). The detailed results are shown in Table 4, aiming to provide a reference for future drug pricing strategies.

**TABLE 3:** The outputs of scenario analysis.

Perspective	ICUR	Overall population		AKT-pathway altered subgroup	
		Without PAP	With PAP	Without PAP	With PAP
the US	5-year horizon	$969,605/QALY	$42,921/QALY	$770,202/QALY	$43,023/QALY
	10-year horizon	***$709,647/QALY***	$70,023/QALY	***$529,726/QALY***	$80,137/QALY
	15-year horizon	$668,006/QALY	$74,151/QALY	$454,756/QALY	$95,116/QALY

Notes: Italicized and bolded data in the table represent base-case outputs. Abbreviation: ICUR, incremental cost-utility ratio; PAP, patient assistance program.

**TABLE 4:** Overview of cost-effective pricing strategies for capivasertib.

Perspective	Threshold of willingness to pay	Time horizon	Overall population	AKT-pathway altered subgroup
the US	$150,000/QALY	5-year horizon	$33.66 per 200 mg	$46.36 per 200 mg
		10-year horizon	$37.35 per 200 mg	$49.66 per 200 mg
		15-year horizon	$38.39 per 200 mg	$48.49 per 200 mg

### Additional analysis

An experimental analysis with specific assumptions in the context of China is also performed. The results and conclusions of this analysis only serve the preset conditions, and the relevant content can be found in Supplementary Content 1.

## Discussion

Breast cancer, one of the leading causes of death among women, poses a significant health threat and places a substantial economic burden on societies worldwide. Ongoing research and the development of innovative anti-cancer drugs have continually introduced more treatment options, striving to mitigate this impact and improve patient survival rates. Capivasertib, as a highly potent pan-AKT kinase inhibitor, has exhibited remarkable therapeutic efficacy when combined with other drugs for advanced breast cancer in previous clinical trials (Andrikopoulou et al., 2022b). However, the feasibility of widespread adoption and clinical application of a drug must consider its economic implications for patients and healthcare systems. Thus, performing an economic assessment of treatment strategies is essential. However, our research fills a crucial gap by providing the first comprehensive economic analysis of capivasertib in combination with fulvestrant regimen alone across different contexts. The absence of similar pharmacoeconomic studies limits the direct comparison of our results with others, underscoring the novelty and importance of our work. Providing the first economic evidence in this analysis, our work can serve as a reference for future pricing and reimbursement decisions. This not only ensures the effective integration of new drugs into clinical practice but also optimizes resource utilization, enhancing overall healthcare outcomes.

The current study aimed to evaluate the cost-effectiveness of adding capivasertib to fulvestrant for patients with HR-positive advanced breast cancer, based on data from the CAPItello-291 trial. Utilizing a partitioned survival model, we found that the inclusion of capivasertib, despite its clinical benefits, does not present a cost-effective treatment option due to its high pricing. Regardless of the overall population group or the AKT pathway-altered subgroup, the ICUR values of the capivasertib combined with fulvestrant regimen versus the fulvestrant regimen alone are all higher than the willingness-to-pay threshold from the perspective of the US. For the experimantal analysis based on specific assumptions from the Chinese perspective, the therapy regimen was also found to lack cost-effectiveness. Our one-way sensitivity analysis revealed that the price of capivasertib significantly impacts the model outcomes. Consequently, we carried out scenario analysis to investigate this further. Our scenario analysis, which extends to different time horizons and prices of capivasertib, found that, from the US perspective, without considering patient assistance programs and excluding Medicare Part D coverage, i.e., with patients fully bearing the cost, the drug price for the overall population group needs to be reduced to $33.66/200 mg to $38.39/200 mg, with a maximum reduction of up to 91.1%, to be potentially cost-effective. For the AKT pathway-altered subgroup, the price needs to be reduced to $46.36/200 mg to $49.66/200 mg, with a reduction of 86.8%–87.7%, to be potentially cost-effective. For the experimental analysis based on specific assumptions from the Chinese perspective, the drug price for the overall population group needs to be reduced to ¥72.58/200 mg to ¥116.91/200 mg, with a reduction of 70.8%–81.9%, to be potentially cost-effective. For the AKT pathway-altered subgroup, the price needs to be reduced to ¥92.05/200 mg to ¥195.56/200 mg, with a reduction of 51.1%–77%, to be potentially cost-effective.

The implications of our findings are substantial for various stakeholders. Pharmaceutical companies might need to reconsider their pricing strategies to enhance the economic viability of capivasertib, especially in markets with stringent cost-effectiveness thresholds. Additionally, our study offers valuable insights for healthcare policymakers and insurance companies regarding the inclusion of capivasertib in reimbursement lists.

Despite the robust methodology, our study has some limitations that warrant consideration. First, the utility values used in our analysis were sourced from published literature rather than directly from the CAPItello-291 trial. Although these values were derived from studies involving similar patient populations, the lack of direct data introduces a degree of uncertainty. Second, the extrapolation of survival curves inherently involves uncertainty. Specifically, converting “time-to-survival” data to “time-to-event” data may introduce biases. Nonetheless, Guyot’s algorithm, widely applied in survival analysis, is recognized for its superior performance in mitigating such biases (Saluja et al., 2019). Another source of uncertainty stems from the selection of survival models. Our one-way sensitivity analysis revealed that the hazard ratio between the treatment regimens significantly influences the ICUR, suggesting that the choice of survival model could substantially impact the results. While we aimed to minimize this impact by selecting the most appropriate survival distribution based on Akaike Information Criterion values and visual inspection, this remains an unavoidable challenge. Additionally, capivasertib is not yet approved in China, and thus, its pricing in our Chinese context analysis was based on empirical assumptions. Given the anticipated approval of capivasertib in the near future, our findings for the Chinese market, derived from hypothetical pricing, might differ from future analyses using actual prices. Nevertheless, we conducted scenario analyses and deduced an economically viable price for the Chinese perspective, considering local economic conditions. This approach helps mitigate the limitation of assumed pricing.

With the continuous evolution of data and the emergence of new evidence, the conclusions of our study may be influenced. Economic analyses should be updated as new clinical and economic data become available. Moreover, it is hoped that more researchers will investigate the cost-effectiveness of capivasertib in various contexts, thereby offering a more comprehensive understanding and supporting informed decision-making for stakeholders.

## Conclusion

In conclusion, while capivasertib in combination with fulvestrant offers significant clinical benefits for HR-positive advanced breast cancer, our study reveals the economic challenges associated with its high pricing. The analysis from the US perspective indicated that the capivasertib plus fulvestrant regimen was not a cost-effective treatment option compared to the fulvestrant regimen alone, regardless of whether it was applied to the overall population or the AKT pathway-altered subgroup. Similarly, in the experimental analysis conducted for China under the assumed pricing conditions, this regimen was also found to lack cost-effectiveness. Future pricing strategies, real-world data, and ongoing economic evaluations will be crucial in determining the broader adoption and reimbursement of this treatment.

## Data Availability

The original contributions presented in the study are included in the article/Supplementary Material, further inquiries can be directed to the corresponding author.
