# Supplementary material for: Adding capivasertib to fulvestrant in patients with hormone receptor-positive advanced breast cancer: a cost-effectiveness analysis
Source: Front Pharmacol. 2025 Jan 15;15:1495082. doi: 10.3389/fphar.2024.1495082 (PMC11774713; doi:10.3389/fphar.2024.1495082)
Supplement: Supplementary file 1 [file DataSheet1.docx]

Supplementary Information

[Supplemental Table 1. Details of model parameter of the analysis for the US perspective. 2](#_Toc184738587)

[Supplemental Table 2．Key model inputs Costs, Utility estimates and other parameters 3](#_Toc184738588)

[Supplemental Table 3. Details of model parameter of the experimental analysis for the Chinese perspective. 4](#_Toc184738589)

[Supplemental Table 4. The outputs of scenario analysis 5](#_Toc184738590)

[Supplemental Table 5. Overview of Cost-Effective Pricing Strategies for Capivasertib 6](#_Toc184738591)

[Supplemental Figure 1. The output of the one-way sensitivity analysis and probabilistic sensitivity analysis. 7](#_Toc184738592)

[Supplementary Content 1: An experimental analysis based on specific assumptions in the context of China 8](#_Toc184738593)

[Methods 8](#_Toc184738594)

[Results 9](#_Toc184738595)

[Conclusion 10](#_Toc184738596)

[Reference 10](#_Toc184738597)

| Supplemental Table 1. Details of model parameter of the analysis for the US perspective. | | | |
| --- | --- | --- | --- |
| parameter | Distribution | Distribution Value 1 | Distribution Value 2 |
| OS HR for Overall patients group | lognormal | meanlog = -0.3105 | sdlog = 0.1374 |
| PFS HR for Overall patients group | lognormal | meanlog = -0.5141 | sdlog = 0.0811 |
| OS HR for AKT pathway-altered subgroup | lognormal | meanlog = -0.3925 | sdlog = 0.2068 |
| PFS HR for AKT pathway-altered subgroup | lognormal | meanlog = -0.7024 | sdlog = 0.1360 |
| cost of capivasertib | normal | mean=377.29 | sd=48.1231 |
| cost of fulvestrant | normal | mean=87.26 | sd=11.1280 |
| cost of administration | normal | mean=702 | sd=89.5425 |
| cost of follow-up | normal | mean=2959 | sd=377.4307 |
| cost of subsequent treatment | normal | mean=2564 | sd=327.0471 |
| cost of End-of-life care | normal | mean=2601 | sd=331.7666 |
| cost of Diarrhea | normal | mean=11545 | sd=1472.5765 |
| cost of Rash | normal | mean=6577.7 | sd=838.9923 |
| cost of Vomiting | normal | mean=3905.7 | sd=498.1760 |
| cost of Anemia | normal | mean=14532 | sd=1853.5714 |
| incidence of Diarrhea in the placebo plus fulvestrant group | beta | α=61.2782 | β=20364.7895 |
| incidence of Rash in the placebo plus fulvestrant group | beta | α=61.2782 | β=20364.7895 |
| incidence of Vomiting in the placebo plus fulvestrant group | beta | α=61.0908 | β=10120.7103 |
| incidence of Anemia in the placebo plus fulvestrant group | beta | α=60.7785 | β=5464.5377 |
| incidence of Diarrhea in the capivasertib plus fulvestrant group | beta | α=55.6563 | β=542.7985 |
| incidence of Rash in the capivasertib plus fulvestrant group | beta | α=53.9073 | β=391.6073 |
| incidence of Vomiting in the capivasertib plus fulvestrant group | beta | α=60.4037 | β=3492.7542 |
| incidence of Anemia in the capivasertib plus fulvestrant group | beta | α=60.2163 | β=2950.5981 |
| utility of PFS | beta | α=15.4630 | β=3.0113 |
| utility of PD | beta | α=55.257 | β=69.4766 |
| Abbreviations: HR hazard ratio, PFS progression-free survival, OS overall survival | | | |

| Supplemental Table 2．Key model inputs Costs, Utility estimates and other parameters | | | |
| --- | --- | --- | --- |
| Parameter | Distribution | China | |
| **Treatment costs** | | **Values (Range)**, CNY | **Reference** |
| Capivasertib (per 200 mg) | Normal | ¥400 (200-600) | Assumed |
| Fulvestrant (per 250 mg) | Normal | ¥2,306 (1,729.5 – 2,882.5) | ^1^ |
| Administration (per cycle) | Normal | ¥225.7 (169.3-282.1) | ^2^ |
| Follow-up | Normal | ¥1,116.5 (837.4-1,395.6) | ^2^ |
| Subsequent treatment | Normal | ¥5,427.9 (4,070.9-6,784.9) | ^2^ |
| End-of-life care | Normal | ¥12,732.5 (9,549.4-15,915.6) | ^2^ |
| **AEs unit costs** | | | |
| Diarrhea | Normal | ¥321.3 (240.9-401.6) | ^3^ |
| Rash | Normal | ¥188.7 (141.5-235.9) | ^4^ |
| Vomiting | Normal | ¥312.2 (234.2-390.3) | ^5^ |
| Anemia | Normal | ¥7,258.1 (5,443.6-9,072.6) | ^6^ |
| **Utility estimates** | | **Values (Range)** | **Reference** |
| PFS state | Beta | 0.87 (0.72 to 0.96) | ^2^ |
| PD state | Beta | 0.71 (0.58 to 0.82) | ^2^ |
| **Disutility estimates** | | **Values (Range)** | **Reference** |
| Diarrhea | Beta | -0.05 (-0.06 to -0.04) | ^7^ |
| Rash | Beta | -0.03 (-0.04 to -0.02) | ^7^ |
| In this table, the costs of AEs presented were paid on a per-event basis. All costs reported for years prior to 2023 are updated to 2023 using the CPI. The average exchange rate between the US dollar and the Chinese yuan is 7.0467 CNY: 1USD. ***Abbreviations:*** ***AEs*** adverse events, ***CNY*** China Yuan (¥). | | | |

| Supplemental Table 3. Details of model parameter of the experimental analysis for the Chinese perspective. | | | | |
| --- | --- | --- | --- | --- |
| parameter | Distribution | Distribution Value 1 | Distribution Value 2 | |
| OS HR for Overall patients group | lognormal | meanlog = -0.3105 | sdlog = 0.1374 | |
| PFS HR for Overall patients group | lognormal | meanlog = -0.5141 | sdlog = 0.0811 | |
| OS HR for AKT pathway-altered subgroup | lognormal | meanlog = -0.3925 | sdlog = 0.2068 | |
| PFS HR for AKT pathway-altered subgroup | lognormal | meanlog = -0.7024 | sdlog = 0.1360 | |
| cost of capivasertib | normal | mean=400 | sd=102.0426 | |
| cost of fulvestrant | normal | mean=2306 | sd=294.1327 | |
| cost of administration | normal | mean=225.7 | sd=28.7883 | |
| cost of follow-up | normal | mean=1116.5 | sd=142.4107 | |
| cost of subsequent treatment | normal | mean=5427.9 | sd=692.3342 | |
| cost of End-of-life care | normal | mean=12732.5 | sd=1624.0434 | |
| cost of Diarrhea | normal | mean=321.3 | sd=40.9821 | |
| cost of Rash | normal | mean=188.7 | sd=24.0689 | |
| cost of Vomiting | normal | mean=312.2 | sd=39.8214 | |
| cost of Anemia | normal | mean=7258.1 | sd=925.7781 | |
| incidence of Diarrhea in the placebo plus fulvestrant group | beta | α=61.2782 | β=20364.7895 | |
| incidence of Rash in the placebo plus fulvestrant group | beta | α=61.2782 | β=20364.7895 | |
| incidence of Vomiting in the placebo plus fulvestrant group | beta | α=61.0908 | β=10120.7103 | |
| incidence of Anemia in the placebo plus fulvestrant group | beta | α=60.7785 | β=5464.5377 | |
| incidence of Diarrhea in the capivasertib plus fulvestrant group | beta | α=55.6563 | β=542.7985 | |
| incidence of Rash in the capivasertib plus fulvestrant group | beta | α=53.9073 | β=391.6073 | |
| incidence of Vomiting in the capivasertib plus fulvestrant group | beta | α=60.4037 | β=3492.7542 | |
| incidence of Anemia in the capivasertib plus fulvestrant group | beta | α=60.2163 | β=2950.5981 | |
| utility of PFS | beta | α=7.1205 | β=1.0640 |  |
| utility of PD | beta | α=17.1150 | β=6.9906 |  |
| Abbreviations: HR hazard ratio, PFS progression-free survival, OS overall survival | | | |  |

| Supplemental Table 4. The outputs of scenario analysis | | | | | |
| --- | --- | --- | --- | --- | --- |
| Perspective | ICUR | Overall population | | AKT-pathway altered subgroup | |
|  |  | without PAP | with PAP | without PAP | with PAP |
| China | 5-year horizon | ¥1,051,430/QALY | ¥477,230/QALY | ¥859,223/QALY | ¥398,519/QALY |
|  | 10-year horizon | ***¥713,500/QALY*** | ¥346,453/QALY | ***¥525,600/QALY*** | ¥271,922/QALY |
|  | 15-year horizon | ¥668,750/QALY | ¥329,066/QALY | ¥436,663/QALY | ¥238,748/QALY |
| Notes: Italicized and bolded data in the table represent base-case outputs. Abbreviation: ICUR incremental cost-utility ratio, PAP patient assistance program | | | | | |

| Supplemental Table 5. Overview of Cost-Effective Pricing Strategies for Capivasertib | | | | |
| --- | --- | --- | --- | --- |
| perspective | threshold of willingness to pay | time horizon | overall population | AKT-pathway altered subgroup |
| China | ¥268,072/QALY | 5-year horizon | ¥72.58 per 200mg | ¥92.05 per 200mg |
|  |  | 10-year horizon | ¥108.76 per 200mg | ¥156.35 per 200mg |
|  |  | 15-year horizon | ¥116.91 per 200mg | ¥195.56 per 200mg |


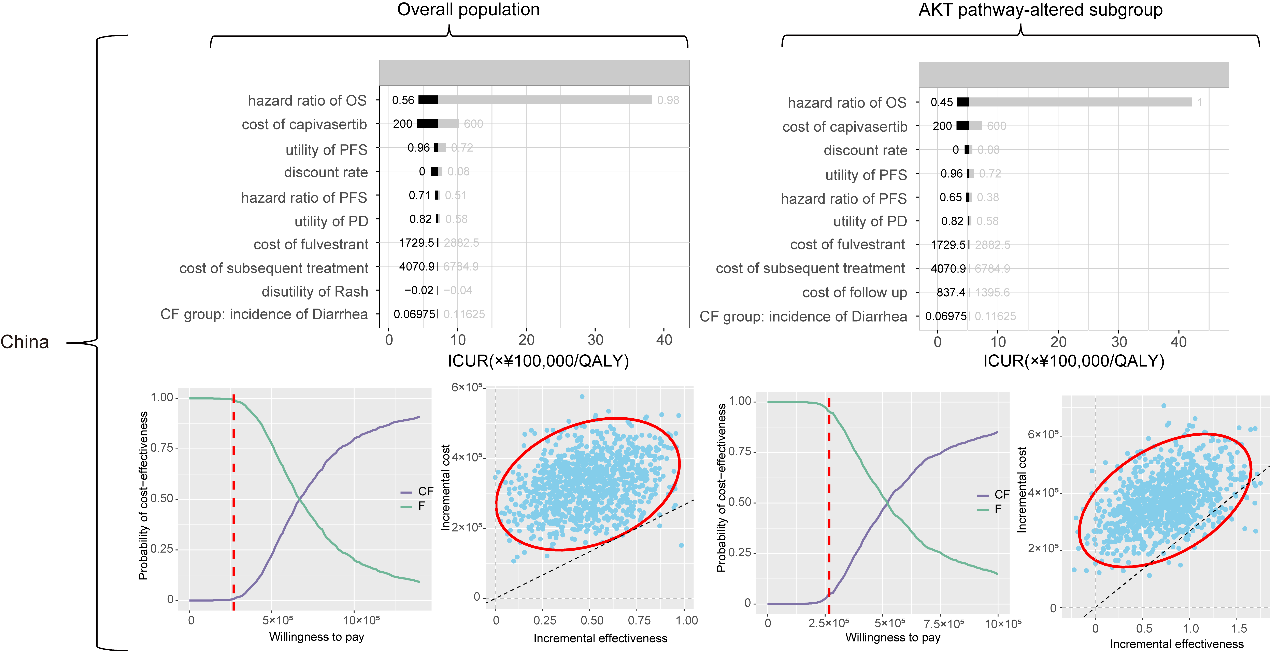


## Supplemental Figure 1. The output of the one-way sensitivity analysis and probabilistic sensitivity analysis.

Notes: In the incremental cost-effectiveness scatter plot, each point represents one output. The red circle denotes the 95% confidence ellipse, while the black dashed line signifies the WTP threshold. In the cost-effectiveness acceptability curve, the y-axis shows the probability that a regimen is cost-effective at different willingness-to-pay thresholds (x-axis). The red dashed line represents the WTP threshold. The monetary unit of the WTP threshold is the Chinese yuan for the analysis in China.

Abbreviations: C chemotherapy, CF capivasertib plus fulvestrant regimen, F fulvestrant regimen alone, PFS progression-free survival, PD progressed disease, ICUR incremental cost-utility ratio, QALY quality-adjusted life-year.

## Supplementary Content 1: An experimental analysis based on specific assumptions in the context of China

### Methods

#### Model structure

This part is the same as the analysis from the perspective of the United States.

#### Treatment regimens and Resource use

This part is the same as the analysis from the perspective of the United States.

#### Clinical data

This part is the same as the analysis from the perspective of the United States.

#### Costs and utilities

This analysis was conducted from the perspectives of healthcare payers in China, focusing primarily on direct medical costs. These expenses expenditures included therapy drugs, intramuscular injection administration, management of severe adverse events (AEs), follow-up care, subsequent treatment and end-of-life care. Drug costs were obtained from a local charge database providing acquisition prices. While expenses related to intravenous injection administration, palliative care, follow-up visits, and subsequent treatment were derived from published studies or databases. The analysis also incorporated costs associated with managing severe AEs, specifically those graded as level 3 or higher.

Health utility played a crucial role in this analysis, serving as the basis for calculating cumulative quality-adjusted life-years (QALYs), a standardized measure of health-related quality of life (HRQOL). It was assumed that the quality of life is tied to the disease progression stages of HR+ advanced breast cancer, including progression-free survival (PFS) and progressed disease (PD). Patients receiving different treatments at the same disease stage were considered to have equivalent health utility values. Additionally, all costs and utilities were discounted, with an annual rate of 5% applied for China. Detailed input values are summarized in Supplemental Table 2.

#### Analyses

This study involves certain assumptions when analyzed from a Chinese healthcare system perspective. Currently, capivasertib has not been approved in China, so there is no existing pricing. A predictive analysis was performed under a specific assumption, where the price of capivasertib was set at ¥400 per 200mg within the analytical model. This assumed price was roughly extrapolated based on the annual treatment costs of several newly marketed antineoplastic agents and was used exclusively for experimental analysis. The conclusions reached are strictly limited to this defined context.

In the base-case analysis, we conducted an assessment of the ICER to determine the additional cost per LY gained between the two treatment regimens. Furthermore, the ICUR was employed to evaluate the additional cost per QALY. A regimen is considered "cost-effective" if the ICUR falls below the specified WTP threshold.

In China, the WTP threshold was determined as three times the per capita gross domestic product (GDP), which amounted to China Yuan (CNY) 268,074 in 2023. This threshold was used as a criterion to evaluate the cost-effectiveness of the treatment regimens within the Chinese context.

We employed a series of uncertainty analyses, including deterministic sensitivity analysis (DSA) and probabilistic sensitivity analysis (PSA), to validate the robustness of results. In the DSA, we examined the impact of individual input uncertainties on the ICUR. The annual discount rate ranged from 0 to 8%, while other model inputs were varied within the reported 95%CI or reasonable ranges (± 25% of the base-case value). Considering that capivasertib is an uncertain factor and we intended to experimentally explore the impact of its values within a larger price range on the results, we set its range to ± 50%. For the PSA, Monte Carlo simulations with 1,000 iterations were conducted, with key parameters sampled based on specified probability distributions. The detailed parameters of these probability distributions were summarized in Supplementary Table 3. To further illustrate the cost-effectiveness of the treatment strategy under varying thresholds, cost-effectiveness acceptability curves (CEAC) and scatter plots were created.

### Results

#### Base-case analysis

For the overall patient population in China, those treated with the fulvestrant regimen gained 2.873 LYs and 1.916 QALYs, incurring an expenditure of ¥232,462. Patients receiving the capivasertib plus fulvestrant regimen achieved 3.606 LYs and 2.372 QALYs, with associated costs of ¥557,818. This indicates an incremental cost of ¥325,356 compared to the fulvestrant regimen alone. The capivasertib plus fulvestrant regimen demonstrated an increase of 0.456 QALYs, and the ICUR was calculated at ¥713,500 per QALY.

For patients with AKT pathway alterations, those on the fulvestrant regimen gained 3.551 LYs and 2.241 QALYs, incurring a cost of ¥273,409. Patients treated with the capivasertib plus fulvestrant regimen experienced gains of 4.699 LYs and 2.931 QALYs, with costs totaling ¥636,073. This resulted in an incremental cost of ¥362,664 and an increase of 0.69 QALYs. The ICUR for the capivasertib plus fulvestrant regimen versus fulvestrant was ¥525,600 per QALY.

#### DSA and PSA

The tornado diagram for the overall patient population showed that the hazard ratio of OS, cost of capivasertib, utility of PFS, discount rate, and hazard ratio of PFS were the key factors significantly impacting the ICUR between the capivasertib plus fulvestrant regimen and the fulvestrant regimen alone. The ICUR ranged from ¥432,901 per QALY to ¥3,825,122 per QALY. In the AKT pathway-altered subgroup, similar sensitivity factors were identified, with the hazard ratio of OS, cost of capivasertib, discount rate, utility of PFS, and hazard ratio of PFS being the main determinants. The ICUR in this subgroup varied from ¥324,358 per QALY to ¥4,209,922 per QALY. In both the overall population and the AKT pathway-altered subgroup, reducing the cost of capivasertib and the hazard ratio of capivasertib plus fulvestrant versus fulvestrant in terms of OS led to a reduction in the ICUR.

The CEAC revealed that in the overall population, the capivasertib plus fulvestrant regimen had approximately a 0.6% probability of being cost-effective at the $268,074/QALY threshold. On the other hand, the fulvestrant regimen alone had a high probability of approximately 99.4% at the same threshold. For the AKT pathway-altered subgroup, the capivasertib plus fulvestrant regimen’s probability of cost-effectiveness increased to 4.8%, while the fulvestrant regimen alone remained highly cost-effective with a probability of around 95.2%. The tornado diagrams, CEACs and scatter plots are shown in Supplemental Figure 1.

#### Scenario analysis

For the analysis from a Chinese perspective, the PAP discount strategy for capivasertib is assumed to set the price at approximately 40% of the initial price. Consequently, we conducted the scenario analysis on time horizon and the pricing of capivasertib to explore potential cost-effective pricing strategies. All results were summarized in the Supplemental Table 4. Furthermore, based on the economic analysis model, we estimated the maximum price at which the drug can achieve cost-effectiveness thresholds (¥268,072/QALY). The detailed results are shown in Supplemental Table 5, aiming to provide a reference for future drug pricing strategies.

### Conclusion

In the experimental analysis conducted for China under the assumed pricing conditions, the capivasertib plus fulvestrant regimen was found to lack cost-effectiveness.

## Reference

1. Chinese Drug Price of Drug Centralized Bid Procurement. Yaozh database. 2024. Accessed March 25, 2024. https://db.yaozh.com/yaopinzhongbiao

2. Zhu W, Zheng M, Xia P, Hong W, Ma G, Shen A. Cost-effectiveness of palbociclib plus fulvestrant as second-line therapy of women with HR+/HER2- advanced breast cancer - A Chinese healthcare system perspective. *Front Oncol*. 2023;13:1068463. doi:10.3389/fonc.2023.1068463

3. Wu B, Ye M, Chen H, Shen JF. Costs of Trastuzumab in Combination With Chemotherapy for HER2-Positive Advanced Gastric or Gastroesophageal Junction Cancer: An Economic Evaluation in the Chinese Context. *Clinical Therapeutics*. 2012;34(2):468-479. doi:10.1016/j.clinthera.2012.01.012

4. Zhou D, Luo X, Zhou Z, et al. Cost-effectiveness analysis of tislelizumab, nivolumab and docetaxel as second- and third-line for advanced or metastatic non-small cell lung cancer in China. *Front Pharmacol*. 2022;13:880280. doi:10.3389/fphar.2022.880280

5. Hurley J, Reis IM, Rodgers SE, et al. The use of neoadjuvant platinum-based chemotherapy in locally advanced breast cancer that is triple negative: retrospective analysis of 144 patients. *Breast Cancer Res Treat*. 2013;138(3):783-794. doi:10.1007/s10549-013-2497-y

6. Dranitsaris G, King J, Kaura S, Yu B, Zhang A. Nab-paclitaxel, docetaxel, or solvent-based paclitaxel in metastatic breast cancer: a cost-utility analysis from a Chinese health care perspective. *CEOR*. Published online May 2015:249. doi:10.2147/CEOR.S82194

7. Nafees B, Stafford M, Gavriel S, Bhalla S, Watkins J. Health state utilities for non small cell lung cancer. *Health Qual Life Outcomes*. 2008;6(1):84. doi:10.1186/1477-7525-6-84

8. Briggs AH, Weinstein MC, Fenwick EAL, et al. Model parameter estimation and uncertainty analysis: a report of the ISPOR-SMDM Modeling Good Research Practices Task Force Working Group-6. *Med Decis Making*. 2012;32(5):722-732. doi:10/f4c4c5
